# Cut-off values for the muscle mass indices determined using DXA in healthy Polish adults – a comparison to EWGSOP2 recommendation

**DOI:** 10.18632/aging.206206

**Published:** 2025-02-26

**Authors:** Aleksandra Radecka, Waldemar Pluta, Tomasz Miazgowski, Anna Lubkowska

**Affiliations:** 1 Department of Functional Diagnostics and Physical Medicine, Pomeranian Medical University in Szczecin, Szczecin 71-210, Poland; 2 Department of Propaedeutic of Internal Diseases and Arterial Hypertension, Pomeranian Medical University in Szczecin, Szczecin 71-252, Poland

**Keywords:** skeletal mass index, DXA, cut-off points, lean body mass

## Abstract

Background: Muscle mass measurements are vital for predicting health outcomes and diagnosing muscle disorders. This study provides reference data for appendicular lean mass (ALM) and total lean mass (TLM) in healthy Polish adults with normal muscle strength and physical performance as per EWGSOP2 guidelines.

Methods: The study included healthy volunteers with normal muscle strength and functional status. Lean mass was measured using Hologic Horizon DXA. Mean values of TLM, ALM, fat-free mass (FFM), and indices (TLMI, ALMI, FFMI) were calculated for seven age groups (by decade). Cut-off points equivalent to T-scores of -1 and -2 standard deviations (SDs) below the young adult reference mean (ages 20-39) were determined.

Results: Data from 1,111 participants (328 men, 46.3 ± 20 years; 783 women, 43.7 ± 23 years) were analyzed. In young adults, mean ALM was 28.1 kg (men) and 17.2 kg (women), and ALMI was 8.6 kg/m^2^ (men) and 6.1 kg/m^2^ (women). Low muscle mass cut-off points (2 SDs below) were 18 kg and 10.9 kg (ALM) and 6 kg/m^2^ and 4.3 kg/m^2^ (ALMI) for men and women, respectively. Men exhibited significantly greater lean mass than women across all age groups (P < 0.001). Lean mass declined with age in both genders, following a nonlinear pattern, except for ALMI in men.

Conclusions: This study provides the first population-based reference values for ALM and TLM in healthy Polish adults aged 20-89 years, integrating criteria for normal muscle strength and physical performance.

## INTRODUCTION

Sarcopenia, classified in the International Classification of Diseases, Tenth Revision, Clinical Modification (ICD-10-CM) code M62.84, is an age-related muscle disease characterized by low skeletal muscle mass and strength combined with low physical performance. This may lead to further impairments in physical mobility and fitness and the subsequent risk of falls, injuries, and fractures. According to the revised diagnostic criteria for sarcopenia recommended by the European Working Group on Sarcopenia in Older People 2 (EWGSOP2) [1], muscle quantity can be reported as total body skeletal muscle mass (SMM), appendicular skeletal muscle mass (ASM), or muscle cross-sectional area of specific muscle groups or various body sites. However, as muscle mass is influenced by body size, when quantifying muscle mass, the absolute value of SMM or ASM should be adjusted for body size using height squared (SSM/ASM to height [2] ratio), weight (SSM/ASM to weight ratio), or body mass index (SSM/ASM to BMI ratio) [1, 2]. Among these indices, the most accurate method with the highest predictive value for identifying subjects at risk for sarcopenia-related clinical implications remains uncertain [1, 3].

Several techniques can be used to measure muscle mass, including dual-energy X-ray absorptiometry (DXA), bioimpedance analysis (BIA), magnetic resonance imaging (MRI), and computed tomography (CT). Although the EWGSOP2 guidelines recommend all these techniques, it is believed that in routine practice, the application of CT and MRI may be limited by the longtime of examination and high costs, CT-generated radiation exposure, and the effect of respiratory motion on image quality for MRI whole-body assessments [3, 4]. DXA is the most widespread technique for measuring body composition [4, 5]. In this method, the use of two different energy spectra is the basis for separately quantifying bone mineral content and the amount of fat tissue and lean tissue. Appendicular lean mass determined by DXA strongly correlated with both MRI (r = 0.88) and CT (r = 0.77–0.95) [5–8]. There are several general pitfalls related to the measurements of muscle mass using CT, MRI, DXA, and BIA [4, 5, 9]. First, there are significant differences between parameters and cut-offs points (CoPs) for SMM assessed by these techniques, and even within the same technique but using devices from different manufacturers. Second, the manufacturer’s reference values have been derived from different populations in terms of age, genetic susceptibility to low muscle mass, nutritional status, ethnicity, comorbidities, and socioeconomic factors. Therefore, it seems advisable that CoPs for body composition components should be determined for narrow populations, thus considering typical anatomical characteristics due to ethnic and racial affiliation, as well as dietary traditions and customs. Moreover, the CoPs may differ regarding the method of parametric normalization (e.g., linear regression or indexing) to account for the body size [9]. Therefore, although various professional associations have published definitions of low SMM [1, 2, 10–12], no consensus definition for low SMM has yet been reached. In the present study, we provide the DXA reference standards for fat-free mass (FFM), lean mass, and muscle mass indices derived from a healthy Polish population aged 20 years or older. We validated these indices against CoPs provided by EWGSOP2 for the European population.

## MATERIALS AND METHODS

### Study design and participants

**Figure 1:**
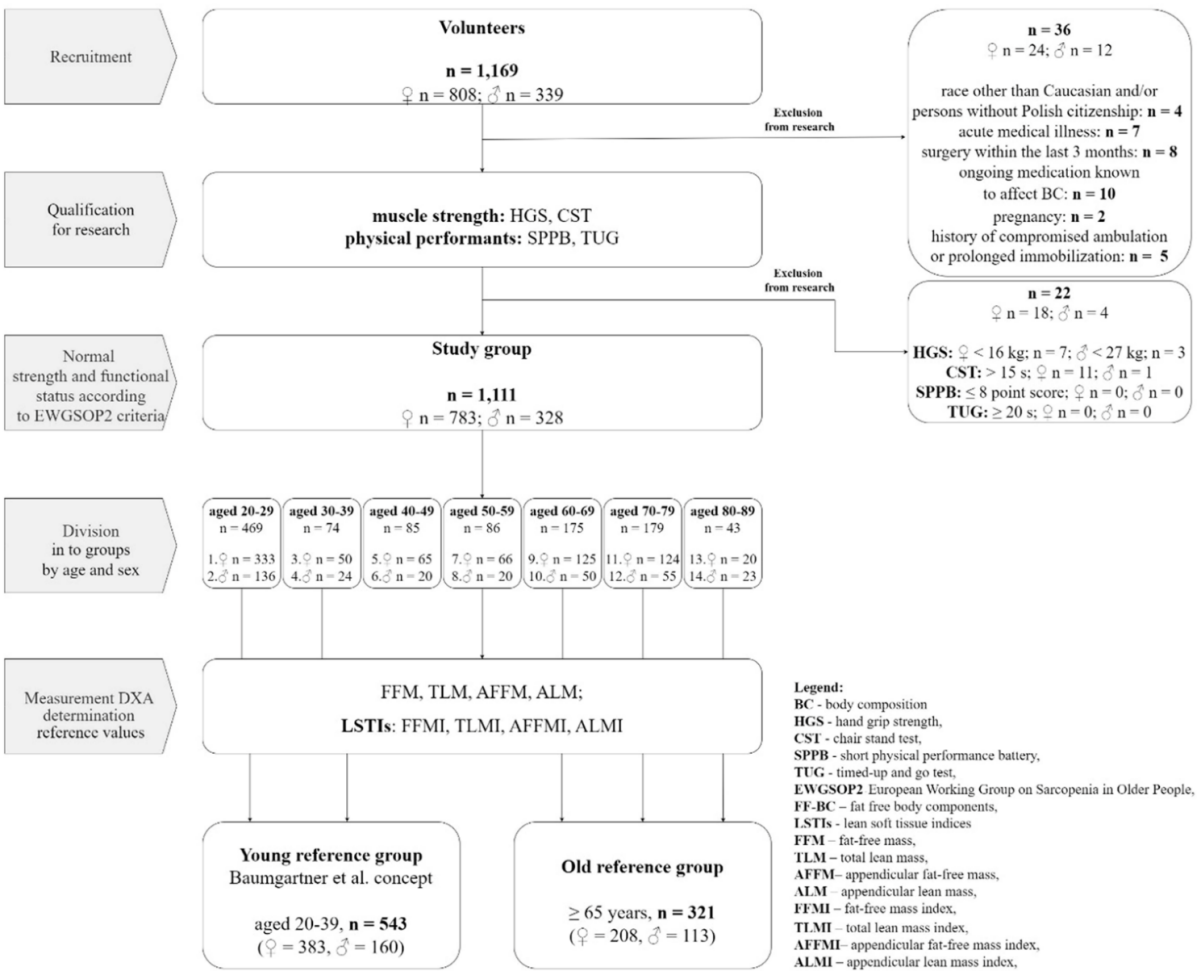
Study flowchart.

This cross-sectional study was conducted at the Department of Functional Diagnostics and Physical Medicine at the Pomeranian Medical University in Szczecin between March 2020 and May 2023. The study complied with all applicable institutional and governmental regulations regarding the ethical use of human volunteers and the terms of the Declaration of Helsinki. The Pomeranian Medical University Bioethics Committee approved the study protocol (KB-0012/146/16-A), and all recruited participants signed their written consent. The study was supported by the research grants obtained from the Pomeranian Medical University (the Science Stimulation Fund No. FSN-318-05/22 and the Statutory Liability Fund No. WNoZ-318/S/2023). The study population was recruited through social media and announcements for university communities, healthcare providers, and institutions promoting healthy lifestyles. The inclusion criteria included the following: 1) age ≥ 20 years; 2) Caucasian race with Polish citizenship and living in Poland since birth; 3) lack of medical conditions requiring chronic pharmacotherapy or other treatments; 4) regular menstruation in premenopausal women; and 5) normal muscle strength and physical performance. We excluded pregnant women and individuals with a history of malignancy, a history of compromised ambulation or prolonged immobilization, surgery within 3 months before evaluation, and taking medications or dietary supplements known to affect body composition parameters. At recruitment, trained personnel carefully reviewed the entry criteria of each volunteer. Overall, 1,169 volunteers were recruited. We excluded 58 participants: 36 did not meet the entry criteria, and the other 22 had suboptimal muscle strength. None of them had abnormal results in physical performance tests. Before statistical analysis, we removed any record elements that could be used to identify participants, such as names, social security numbers, addresses, and ID numbers. The study’s flowchart is shown in Figure 1.

Muscle strength and physical performance tests were used as initial tests, aiming to exclude individuals at risk of sarcopenia. Muscle strength was assessed by the Hand Grip Strength Test (HGS) and the Chair Stand Test (CST). Physical performance was evaluated using a Short Physical Performance Battery (SPPB) and a Timed-Up and Go (TUG) test. All tests were performed using a standard protocol and CoPs recommended by EWGSOP2 for sarcopenia: HGS < 16 kg in females and < 27 kg in males, CST > 15 seconds, SPPB score ≤ 8 points, and TUG ≥ 20 seconds [1].

### Anthropometry and DXA-derived body composition

Body weight and height were measured using a digital scale and an electronic stadiometer. Body composition was assessed using the Hologic Horizon DXA System (Quirugil, Bogota, Colombia) with Discovery software version 12.3 (Bellingham, WA, USA). According to the manufacturer’s protocol, the device was calibrated daily using a spinal phantom and in the extended version every 8 days. The calibration results were analyzed for parameter variation to monitor the stability and accuracy of the measurements. In addition, the device is serviced annually by a certified technical team to ensure the quality of the results and measurements obtained. Preparation of the test subject: maintaining at least a 10–12-hour break from meals and energy stimulants, including coffee, 24 hours of abstinence from taking calcium supplements and undertaking intense physical activity. The need to urinate before the test, and to remain calm and assume an appropriate position during the test [13]. For the examination, the volunteer removed her clothing as well as all jewellery, remaining only in metal-free underwear. During the study, the volunteer stayed motionless in a strictly fixed position according to the NHANES method (palms down, hands isolated from the body, feet neutral, arms straight or slightly stooped, face up with a neutral chin) [14]. A single, trained technician analyzed all DXA scans. Automatic scan mode and automatic analysis mode were used as the default settings. Anthropometric and body composition data were used to obtain total lean mass (TLM), fat mass, and bone mineral content. In DXA, TLM is a measure of all non-fat lean tissue, including muscles, internal organs, and connective tissue, but does not include bone mineral content. FFM, in turn, includes lean soft tissue and bones. Although DXA-derived TLM is not a direct measure of muscle mass, it is believed that it can be used as a proxy for skeletal muscles in humans [4, 15]. Appendicular lean mass (ALM) and appendicular fat-free mass (AFFM) were estimated as the sum of the lean and fat tissue in the arms and legs, respectively. All parameters were adjusted by height squared to obtain the following lean soft tissue indices (LSTIs): appendicular lean mass index (ALMI; ALM/Height^2^); appendicular fat-free mass index (AFFMI; AFFM/Height^2^); total lean mass index (TLMI; TLM/Height^2^); and total fat-free mass index FFMI (FFM/Height^2^).

### Statistical analysis

The data are presented as the group mean ± standard deviation (SD). The distribution of men and women between decades was checked for normality using the Shapiro-Wilk test. This distribution was found to be non-normal. Sex- and age-specific absolute FFM, AFFM, TLM, and ALM, as well as LSTIs, were calculated, with participants classified into 7 age groups by decade (20–29, 30–39, 40–49, 50–59, 60–69, 70–79, and ≥80). The CoPs were calculated using healthy young adult (aged 20–39 years) reference data and were equal to a T score of −2.0 and −1.0 (the number of SDs below the young adult reference mean). Statistical differences between women and men were evaluated using the Student’s t-test for independent samples. One-way ANOVA was used to compare obtained outcome variables between age groups. The relationship between age and measured muscle variables was assessed using regression analyses. Since linear models did not satisfy the assumptions of linear regression models, we used polynomial regression. McNemar’s test was used to compare the rates of low muscle mass defined as a T-score of ≤ -2 SDs below the young reference group in our sample and the EWGOSP2 recommendations. Relationships between age and covariates were displayed as scatterplots with 95% prediction intervals. An alpha level was set at 0.05 to determine statistical significance. Statistical analyses were performed using Statistica PL software version 13.3 (StatSoft, Cracow, Poland).

### Data availability statement

The datasets used and/or analysed during the current study are available from the corresponding author on reasonable request.

## RESULTS

### Participants

The characteristics of all participants (ages 20 to 89 years) are presented in Table 1. Mean BMI and DXA-derived lean parameters were higher in men than women. Men also had significantly higher scores on the HGS test and shorter time at CST and TUG, while the SPPB scores were comparable in both genders.

**Table 1:** Participant characteristics.

	**All (n = 1,111)**		**Women (n = 783)**		**Men (n = 328)**		***P*-value**
	**Mean**	**SD**	**Mean**	**SD**	**Mean**	**SD**	
Age (years)	44.48	22.21	43.71	21.80	46.33	23.09	0.072
Weight (kg)	72.21	15.54	67.18	13.13	84.23	14.21	0.001
Height (cm)	168.5	9.59	164.8	7.57	177.3	8.09	0.001
BMI (kg/m^2^)	25.36	4.82	24.72	4.85	26.89	4.39	0.001
FFM (kg)	48.13	11.19	42.64	6.28	61.23	9.31	0.001
TLM (kg)	45.80	10.82	40.50	6.08	58.45	9.00	0.001
AFFM (kg)	20.92	5.92	18.08	3.36	27.69	5.18	0.001
ALM (kg)	19.70	5.65	17.00	3.21	26.13	4.99	0.001
FFMI (kg/m^2^)	16.82	2.98	15.70	2.39	19.49	2.54	0.001
TLMI (kg/m^2^)	16.01	2.91	14.92	2.33	18.60	2.47	0.001
AFFMI (kg/m^2^)	7.27	1.52	6.63	1.07	8.79	1.36	0.001
ALMI (kg/m^2^)	6.84	1.47	6.24	1.03	8.29	1.32	0.001
HGS (kg)	30.78	10.5	26.63	5.28	45.77	11.03	0.001
CST (s)	8.40	3.75	8.61	3.84	7.41	3.15	0.031
TUG (s)	5.64	1.59	5.77	1.59	5.02	1.47	0.001
SPPB (score)	11.27	1.12	11.24	1.16	11.42	0.94	0.273

*P*-value refers to comparisons between men and women. BMI, body mass index; FFM, fat-free mass; TLM, total lean mass; AFFM, appendicular fat-free mass; ALM, appendicular lean mass; FFMI, fat-free mass index; TLMI, total lean mass index; AFFMI, appendicular fat-free mass index; ALMI, appendicular lean mass index; HGS, hand grip strength; CST, Chair Stand Test; TUG, Timed-Up and Go test; SPPB, Short Physical Performance Battery.

### Reference data for young, healthy adults

Young adult (20–39 years) reference data for lean mass parameters and LSTIs and CoPs equivalent to T-scores of –1 and –2 SDs are presented in Table 2. Absolute values of FFM, TLM, AFFM and ALM, as well as values of corresponding LSTIs, were significantly greater in males compared to women at *P* < 0.001. The reference values for absolute ALM were 28.1 kg in men and 17.2 kg in women, while for ALMI, they were 8.6 kg/m^2^ and 6.1 kg/m^2^ in men and women, respectively. The CoPs defining a low muscle mass (2 SDs below the young adult reference mean) in sarcopenia according to the current definition [1] were 10.9 kg in women and 18 kg in men for ALM, and 4.3 kg/m^2^ in women and 6 kg/m^2^ in men for ALMI.

**Table 2:** Sex-specific mean values and T-scores of lean components for the reference group of healthy young adults (20–39 years).

	**Females (n = 383)**			**Males (n = 160)**		
	**Mean ± SD**	**–1 SD**	**–2 SD**	**Mean ± SD**	**–1 SD**	**–2 SD**
FFM (kg)	42.24 ± 6.61	36.03	29.82	62.97 ± 9.33	54.0	44.0
TLM (kg)	39.98 ± 6.01	33.99	27.99	60.07 ± 9.03	51.0	42.0
AFFM (kg)	18.34 ± 3.30	15.04	11.74	29.66 ± 5.13	25.0	19.0
ALM (kg)	17.22 ± 3.16	14.06	10.90	28.05 ± 4.94	23.0	18.0
FFMI (kg/m^2^)	15.02 ± 1.76	13.27	11.51	19.25 ± 2.42	17.0	14.0
TLMI (kg/m^2^)	14.22 ± 1.71	12.51	10.80	18.37 ± 2.35	16.0	14.0
AFFMI (kg/m^2^)	6.51 ± 0.95	5.56	4.61	9.06 ± 1.32	8.0	6.0
ALMI (kg/m^2^)	6.12 ± 0.92	5.20	4.27	8.57 ± 1.27	7.0	6.0

*P*<0.001 for all comparisons between males and females. FFM, fat-free mass; TLM, total lean mass; AFFM, appendicular fat-free mass; ALM, appendicular lean mass; FFMI, fat-free mass index; TLMI, total lean mass index; AFFMI, appendicular fat-free mass index; ALMI, appendicular lean mass index.

### Sex-specific reference values for lean mass indices by age

**Figure 2:**
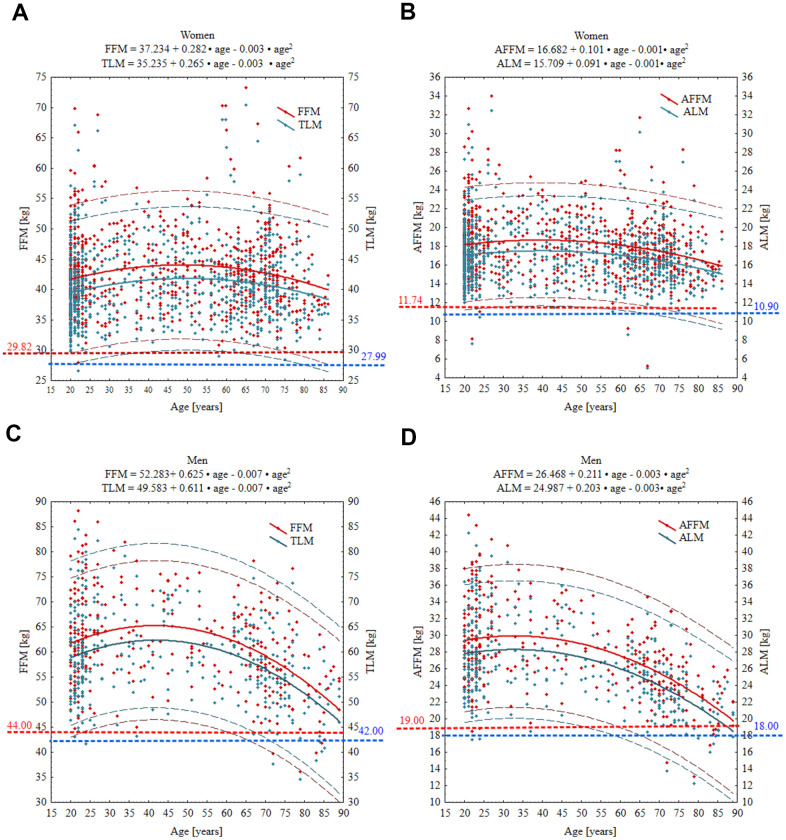
Age-specific curves for FFM, TLM, AFFM and ALM in women (**A**, **B**) and men (**C**, **D**). Wide solid line, the regression line; narrow dashed line, 95% prediction interval; horizontal spotted line, cut-off point equivalent to T-score of –2 SD. Red and blue numbers denote the absolute value of given parameter at T-score = –2. Abbreviations: FFM, fat free mass; TLM, total lean mass; AFFM appendicular fat free mas; ALM, appendicular lean mass.

Mean (± SD) and T-scores for FFM, TLM, AFFM, and ALM from the Hologic Horizon-DXA models by age for both women and men are shown in Table 3. Men had significantly greater all lean mass parameters than women (*P* < 0.001) across all age groups. Mean FFM, AFFM, and TLM were the greatest in 40–49-year-old men and decreased with increasing age, particularly in the two oldest age groups. In women, all these measures remained relatively steady across all age groups, except the 8^th^ decade, in which they were significantly reduced (*P* < 0.05 vs. 20–39 years). ALM progressively decreased starting in the 4^th^ decade both in men and women, and in the 8^th^ decade, it was reduced by 11% (women) up to 23% (men) compared to the youngest volunteers. All absolute values of FFM, TLM, AFFM, and ALM showed curvilinear relationships with age. These relationships are displayed in Figure 2 as scatterplots with 95% prediction intervals together with polynomial regression equations.

**Table 3:** Sex-specific mean values and T-scores of lean components by 10-year age groups.

**Age group (years)**	**Sex, n**	**FFM (kg)**			**TLM (kg)**			**AFFM (kg)**			**ALM (kg)**		
		**Mean ± SD**	**T-score**		**Mean ± SD**	**T-score**		**Mean ± SD**	**T-score**		**Mean ± SD**	**T-score**	
			**–1**	**–2**		**–1**	**–2**		**–1**	**–2**		**–1**	**–2**
20-29	F = 333	42.05 ± 6.2	35.83	29.61	39.81 ± 6.0	33.80	21.78	18.31 ± 3.3	14.99	11.68	17.18 ± 3.1	14.01	10.84
	M = 136	62.78 ± 9.2	53.51	44.24	59.87 ± 8.9	50.88	32.90	29.68 ± 5.1	24.56	19.44	28.05 ± 4.9	23.12	18.19
30-39	F = 50	43.48 ± 6.0	37.43	31.38	41.17 ± 5.8	35.31	23.59	18.58 ± 3.2	15.35	12.12	17.46 ± 3.1	14.34	11.22
	M = 24	64.01 ± 9.7	54.24	44.47	61.21 ± 9.4	51.81	33.01	29.60 ± 5.3	24.27	18.94	28.04 ± 5.1	22.94	17.84
40-49	F = 65	43.53 ± 5.4	38.11	32.69	41.21 ± 5.2	35.96	25.46	18.51 ± 2.7	15.73	12.96	17.34 ± 2.6	14.69	12.04
	M = 20	66.34 ± 8.0^a^	58.29	50.25	63.27 ± 7.8^a^	55.43	47.58	30.16 ± 4.3^a^	25.83	21.50	26.54 ± 4.2^a^	24.31	20.08
50-59	F = 66	43.58 ± 6.8	36.78	29.98	41.41 ± 6.5	34.85	21.73	18.35 ± 3.2	15.16	11.97	17.23 ± 3.1	14.17	11.11
	M = 20	61.72 ± 6.4	55.31	48.90	58.99 ± 6.2	52.73	40.21	27.89 ± 3.2	24.67	21.45	26.34 ± 3.1	23.23	20.12
60-69	F = 125	43.02 ± 7.1	35.92	28.82	41.08 ± 6.9	34.18	20.38	17.91 ± 4.4	13.48	9.06	16.88 ± 4.2	12.66	8.44
	M = 50	61.91 ± 6.7	55.18	48.46	59.14 ± 6.5	52.63	39.61	26.65 ± 3.2^b^	23.44	20.23	25.08 ± 3.1^b^	22.02	18.96
70-79	F = 124	43.14 ± 5.8	37.26	31.39	41.19 ± 5.7	35.49	29.80	16.43 ± 2.5^a^	13.90	11.38	16.45 ± 2.6^a^	14.82	12.20
	M = 55	56.93 ± 8.0^b^	48.89	40.85	54.42 ± 7.7^b^	46.63	31.05	24.08 ± 3.6^b^	20.42	16.76	22.66 ± 3.5^b^	19.16	15.66
≥ 80	F = 20	38.86 ± 3.2^a^	35.65	32.44	37.25 ± 3.19^a^	34.06	27.68	16.08 ± 1.7^a^	14.30	12.52	15.28 ± 1.7^a^	13.52	11.76
	M = 23	51.64 ± 6.5^b^	45.08	38.52	49.21 ± 6.24^b^	42.97	30.49	21.82 ± 2.7^b^	19.12	16.42	20.48 ± 2.5^b^	17.95	15.42

^a^*P*<0.05, ^b^*P*<0.001 in comparison to healthy young adults (20–39 years). FFM, fat-free mass; TLM, total lean mass; AFFM, appendicular fat-free mass; ALM, appendicular lean mass.

Mean values of FFMI, TLMI, AFFMI and ALMI, and CoPs equivalent to T-scores of –1.0 and –2 SDs are presented in Table 4. FFMI in women increased progressively from 14.95 kg in the youngest age group to 16.7 kg in the 70–79 years group. In the oldest group (≥80 kg), FFMI decreased but was still slightly higher in comparison to the youngest group. In contrast, FFMI in men was relatively steady across all ages. TLMI, in turn, was the lowest in women from the young reference group, while starting from the 4^th^ decade, remained at a slightly but significantly higher level across older age decades. In opposition to women, TLMI in men was the greatest in the 4^th^ and 5^th^ decades and then comparable with the youngest age groups. AFFMI value in women was significantly higher in 60–69 years old compared to the other groups, while in men starting from 60–69 years old, it showed a significant decreasing trend. A similar pattern to AFFMI was also observed in ALM performance. The prevalence of low muscle mass, defined as a *T*-score of ≤ -2 SDs below the young reference group, ranged from 0.13% to 0.89% in women and from 0.3% to 3.05% in men (Table 5). The prevalence of muscle mass loss of 1 SD to 2 SDs ranged from 7.8% to 15.2% in women and from 11.3% to 31.7% in men, depending on the lean mass parameter used for assessment.

**Table 4:** Sex-specific mean values and T-scores of muscle mass indices by 10-year age groups.

Age group (years)	Sex, n	FFMI (kg/m^2^)			TLMI (kg/m^2^)			AFFMI (kg/m^2^)			ALMI (kg/m^2^)		
		Mean ± SD	T-score		Mean ± SD	T-score		Mean ± SD	T-score		Mean ± SD	T-score	
			–1	–2		–1	–2		–1	–2		–1	–2
20–29	F = 333	14.95 ± 1.73	13.22	11.49	14.15 ± 1.69	12.46	10.77	6.51 ± 0.95	5.55	4.60	6.10 ± 0.91	5.19	4.28
	M = 136	19.12 ± 2.40	16.72	14.32	18.23 ± 2.34	15.89	13.55	9.03 ± 1.33	7.70	6.37	8.53 ± 1.29	7.24	5.95
30–39	F = 50	15.49 ± 1.85	13.64	11.79	14.67 ± 1.81	12.87	11.07	6.61 ± 0.99	5.62	4.63	6.21 ± 0.96	5.25	4.29
	M = 24	20.03 ± 2.42	17.61	15.19	19.15 ± 2.33	16.82	14.49	9.24 ± 1.26	7.98	6.72	8.75 ± 1.21	7.55	6.35
40–49	F = 65	15.76 ± 2.11^a^	13.66	11.56	14.92 ± 2.03^a^	12.89	10.86	6.68 ± 0.96	5.72	4.76	6.26 ± 0.91	5.35	4.44
	M = 20	20.56 ± 2.78^a^	17.79	15.01	19.61 ± 2.69^a^	16.92	14.23	9.33 ± 1.28	8.05	6.76	8.83 ± 1.29	7.54	6.25
50–59	F = 66	15.93 ± 2.21^a^	13.72	11.51	15.13 ± 2.14^b^	12.99	10.85	6.71 ± 1.09	5.70	4.70	6.29 ± 0.96	5.33	4.37
	M = 20	19.95 ± 2.11	17.84	15.73	19.07 ± 2.06	17.01	14.95	8.93 ± 0.98	7.95	6.97	8.43 ± 0.95	7.48	6.53
60–69	F = 125	16.74 ± 3.71^c^	12.93	9.22	15.89 ± 3.59^c^	12.30	8.71	6.87 ± 1.58^a^	5.29	3.71	6.48 ± 1.51^b^	4.97	3.46
	M = 50	19.83 ± 1.75	18.08	16.33	18.95 ± 1.73	17.22	15.49	8.53 ± 0.84^b^	7.69	6.85	8.03 ± 0.82^a^	7.21	6.39
70–79	F = 124	16.68 ± 2.02^c^	14.66	12.64	15.82 ± 1.97^c^	13.95	11.98	6.72 ± 0.89	5.83	4.95	6.33 ± 0.86^a^	5.46	4.60
	M = 55	19.25 ± 2.50	16.75	14.25	18.39 ± 2.43	15.96	13.53	8.11 ± 1.20^c^	6.91	5.71	7.63 ± 1.15^c^	6.48	5.33
≥ 80	F = 20	15.88 ± 1.68	14.20	12.52	15.22 ± 1.65^a^	13.57	11.92	6.54 ± 0.73	5.81	5.08	6.22 ± 0.71	5.51	4.80
	M = 23	19.07 ± 2.18	16.89	14.71	18.19 ± 2.14	16.05	13.91	8.25 ± 1.50^b^	6.75	5.25	7.77 ± 1.51^c^	6.26	4.75

^a^*P*<0.05, ^b^*P*<0.01, ^c^*P*<0.001 in comparison to healthy young adults (20–39 years). FFMI, fat-free mass index; TLMI, total lean mass index; AFFMI, appendicular fat-free mass index; ALMI, appendicular lean mass index.

**Figure 3:**
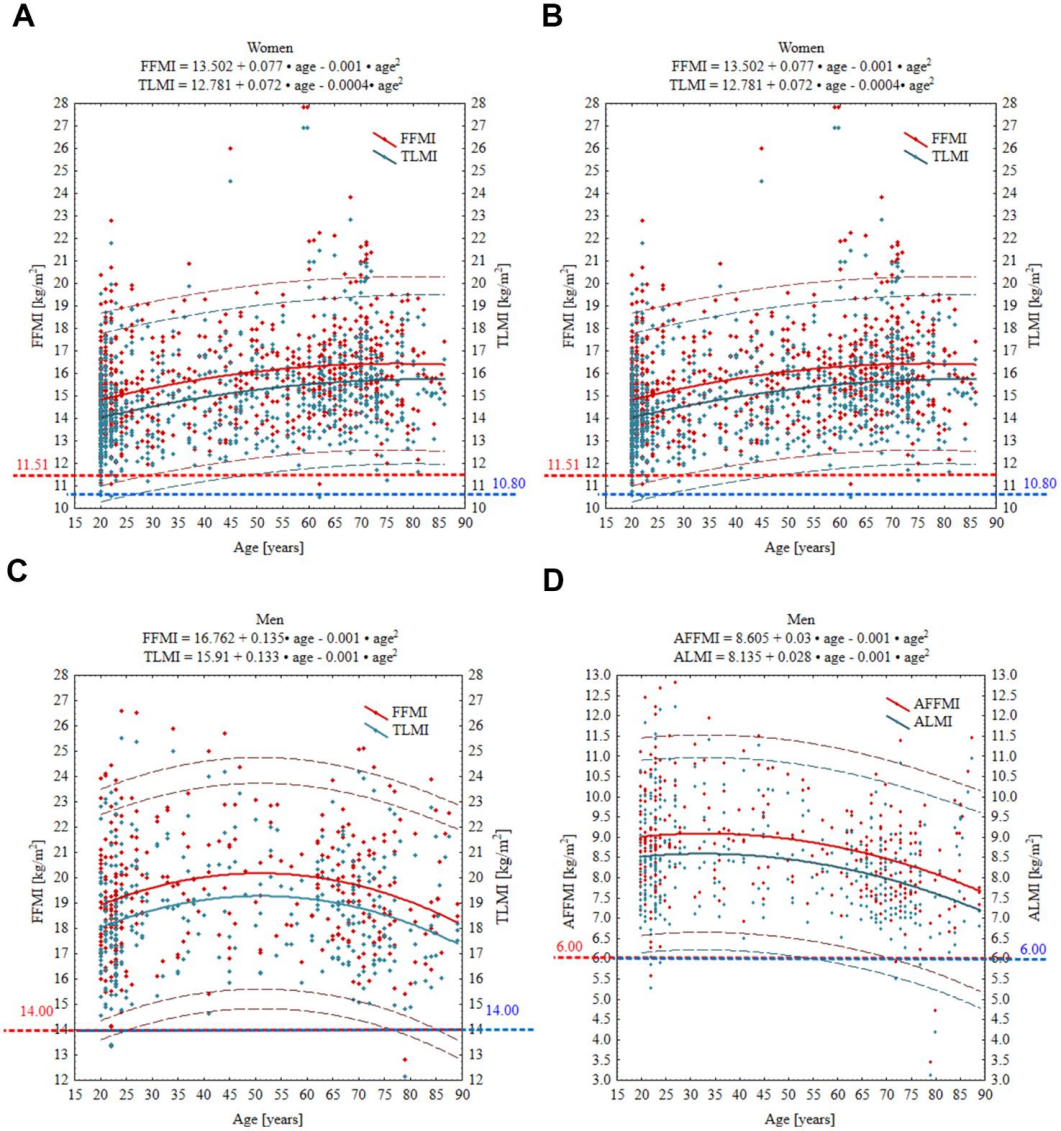
Age-specific curves for FFMI, TLMI, AFFMI and ALMI in women (**A**, **B**) and men (**C**, **D**). Wide solid line, the regression line; narrow dashed line, 95% prediction interval; horizontal spotted line, cut-off point equivalent to T-score of –2 SD. Red and blue numbers denote the absolute value of given parameter at T-score = –2. FFMI, fat free mass index; TLMI, total lean mass index; AFFM appendicular fat free mass index; ALMI, appendicular lean mass index.

However, when we applied the updated EWGSOP2 CoPs, the rates of low muscle mass were significantly higher (P < 0.001) both in women (20.3-25.6%) and men (8.5-11.6%) (Table 5). The relationships between age and TLM, and ALM were negative and nonlinear. In women, age was significantly associated with ALM (*P* = 0.007), TLM (*P* < 0.001), ALMI (*P* = 0.012), and TLMI (*P* < 0.001), while in men, age was significantly associated with ALM, TLM, and TLMI (all *P* < 0.001) but not with ALMI. These relationships are displayed in Figures 2, 3 as scatterplots with 95% prediction intervals along with the relevant regression equations.

**Table 5:** Prevalence of lean mass parameters within defined T-score categories for men and women.

**Lean soft tissue parameters**	**Polish population**		**EWGOSP 2 n (%)**
	**T-score category n (%)**		
	**–1 SD**	**–2 SD**	
Women (n = 783)			ALM < 15kg ALMI < 5.5 kg/m^2^
FFM (kg)	99 (12.64)	1 (0.13)	
AFFM (kg)	119 (15.20)	4 (0.51)	
TLM (kg)	99 (12.64)	1 (0.13)	
ALM (kg)	116 (14.81)	4 (0.51)	200 (25.6)^a^
FFMI (kg/m^2^)	61 (7.79)	4 (0.51)	
AFFMI (kg/m^2^)	70 (8.94)	7 (0.89)	
TLMI (kg/m^2^)	64 (8.17)	4 (0.51)	
ALMI (kg/m^2^)	68 (8.68)	6 (0.77)	159 (20.3)^a^
Men (n = 328)			ALM < 20kg ALMI < 7.0 kg/m^2^
FFM (kg)	69 (21.04)	7 (2.13)	
AFFM (kg)	104 (31.71)	8 (2.44)	
TLM (kg)	65 (19.82)	7 (2.13)	
ALM (kg)	88 (26.83)	10 (3.05)	28 (8.54)^a^
FFMI (kg/m^2^)	44 (13.41)	1 (0.30)	
AFFMI (kg/m^2^)	97 (29.57)	4 (1.22)	
TLMI (kg/m^2^)	37 (11.28)	5 (1.52)	
ALMI (kg/m^2^)	38 (11.59)	7 (2.13)	38 (11.59)^a^

Results of McNemar’s test ^a^ P<0.001 significance of differences between the occurrence of low muscle mass in the study group according to CoPs developed for healthy young (20–39 years) adults and CoPs recommended by EWGOSP 2. FFM, fat-free mass; AFFM, appendicular fat-free mass; TLM, total lean mass; ALM, appendicular lean mass; FFMI, fat-free mass index; AFFMI, appendicular fat-free mass index; TLMI, total lean mass index; ALMI, appendicular lean mass index.

## DISCUSSION

Low muscle mass is an independent and significant risk factor for falls, fractures, weakness, morbidity, and even mortality in the elderly. Therefore, the need to determine objective CoP indicators of low muscle mass is raised more and more often, and more and more attention is paid to the occurrence of sarcopenia in the elderly [16–18].

In this report, we provide the first reference standards for lean mass indices in healthy Polish adults aged 20–89 years using the Hologic Horizon-DXA system. Based on the results of previously performed strength and physical performance tests recommended by the latest EWGSOP2 guidelines, all subjects with the risk of sarcopenia were excluded from the study. We present our data in absolute mass units, mass adjusted for height squared, and *T*-scores in 10-year age groups, separately for men and women. These reference values, when used in combination with measurements of muscle strength and physical performance, have important clinical applications, particularly in the early diagnosis and management of sarcopenia. Alone or combined with measurements of muscle strength and physical performance, these data may be useful for assessing pre-sarcopenia and sarcopenia in the adult population above 20 years. It is worth noting that this is only the second such large data (1,111 participants) for the European population on measurements of lean mass indices calculated from a whole-body scan by the DXA method. The first Copenhagen study was performed on a Danish cohort aged 20-93 years in a group of 1,305 participants [17].

Sarcopenia is determined by the interaction of genetic and environmental factors, and the key indicator differentiating the phenotype is low muscle mass. Studies confirm that the incidence of the disease varies significantly between populations, which is why a correct diagnosis should be based on very specific reference values [19]. An example is a study of a multi-ethnic population in Singapore, which found that sarcopenia affected 27.4 per cent of older people with type 2 diabetes, indicating the need to consider local reference standards when assessing risk [20]. Ethnic differences are further highlighted in the meta-analysis, which suggests that sarcopenia is more common in Asian populations with lower BMI, highlighting the importance of localised diagnostic criteria [21]. In Malaysia, lifestyle factors such as dietary habits and level of inflammation are significant predictors of sarcopenia risk, suggesting the need to tailor interventions to population specificity [22]. In addition, studies on Korean cohorts have identified specific genetic variants that may affect muscle mass, highlighting the need to consider genetic differences in the diagnosis and treatment of sarcopenia [23]. Our study provides such region-specific reference values, which can better reflect the body composition of the Polish population compared to European-wide standards.

Published reference standards for muscle mass differ widely depending on the outcome parameter and reference population. In addition to known inter- and intra-assay variations between methods used for the assessment of muscle mass, a vast majority of reference values derive from the general population, including a multiethnic large National Health and Nutrition Examination Survey (NHANES) dataset comprising black, white, and Mexican Americans [24, 25], standards for American adults [26], Hispanic and non-Hispanic residents of New Mexico [27], residents of southeastern Australia [28], community-dwelling adults in Australia [29], and Chinese [30] and Singaporean adults [31]. All these reference values use CoPs from young adults to identify older subjects with low muscle mass. However, the general population includes cases with known and unknown comorbidities, including impaired muscle fitness. In addition, the prevalence of sarcopenia varies depending on the geographical region and setting of the population sampled. The prevalence of sarcopenia in the general population was estimated at approximately 10-13% [18, 22], but the rates increase with age, and at the age of 80, it may reach even 50% [22]. It is also important that DXA-based lean soft tissue parameters are influenced by ethnicity and the amount of fat mass [2, 4, 9, 32, 33]. Therefore, the arbitrarily set CoPs for muscle mass derived from the general population may not be applicable to all geographical regions, ethnicities, and settings of the population sampled [17, 18]. This underscores the importance of developing regional standards that reflect the specific body composition and health profiles of the population in question, such as the Polish cohort in our study.

In an alternative approach (also called a normative approach), suitable reference values require an appropriate sample size, ideally comprised of healthy individuals [9, 32]. Such an approach might be helpful in the future determination of *Z*-scores that compare the patient’s individual raw result with healthy controls matched for sex, ethnicity, and age. It may also facilitate the comparison of results between studies. Therefore, it seems justifiable to develop age- and sex-specific CoPs for muscle mass in healthy individuals, including those with normal muscle fitness. We applied this approach to our inclusion criteria and selected a sample of healthy subjects with normal scores on HGS, CST, SPPB, and TUG. By ensuring that the reference population consisted of individuals with normal muscle strength and physical performance, we aimed to provide clinically relevant cut-off points that can be more accurately applied in the diagnosis and management of sarcopenia. Noteworthy, using such rigorous inclusion criteria, we found 22 (2%) participants with impaired muscle strength in our sample. The first European standards for DXA-lean soft tissue indices derived from 1,305 healthy volunteers aged 20–93 years were developed in 2019 in the frame of the Copenhagen Sarcopenia Study (CSS) [17]. Despite some methodological differences between CSS and our study (e.g., in contrast to our study, low muscle strength was not an exclusion criterion in CSS, and both studies used the DXA devices coming from two different manufacturers) and differences in participant characteristics (our cohort was older and had slightly higher BMI), the mean ALMI values in the reference age group (20–39 years) were similar (8.6 vs. 8.5 kg/m^2^ and 6.6 vs. 6.1 kg/m^2^ for men and women, in CSS and Polish sample, respectively). The mean ALM values in our sample were also comparable to those in CSS. However, when our *T*-scores at –2.0 SDs were compared with those in CSS, the differences between both cohorts were greater. The *T*-score defining a low ALM in CSS was 21 kg in men and 13.2 kg in women, while in our cohort, it was 18 kg and 11 kg, respectively. Similarly, a *T*-score of –2 SDs defining a low ALMI in CSS was set at a higher level than in our study (6.6 vs. 6 kg/m^2^ in men and 5.0 vs. 4.3 kg/m^2^ in women). Despite these discrepancies, our mean ALMI values at *T*-score equal –2 SDs were lower than those proposed by EWGSOP [1].

These differences between the Polish and Danish cohorts highlight the need for region-specific reference standards, as using European-wide or international cut-off points may lead to misclassification in certain populations. For example, the lower cut-off values observed in our study compared to CSS suggest that Polish adults may have slightly different body composition profiles, which must be considered when diagnosing sarcopenia. Developing regional standards, like those provided in this study, ensures that clinicians can make more accurate assessments of sarcopenia risk based on the specific characteristics of their patient populations.

On the other hand, some studies reported much higher muscle mass in the 20–39-year-old reference group compared to our results and results from CSS. In standards developed by Imboden et al. [26] for US Caucasian adults using GE Healthcare Lunar Prodigy, the mean ALMI was 8.9–9.5 kg/m^2^ in men and approximately 7.0 kg/m^2^ in women. Similar values were also reported in the Australian Body Composition (ABC) study [29].

In line with earlier reports [17, 22, 24, 26, 31, 34, 35], the present study demonstrated greater lean mass parameters in men than women for all age groups (Tables 3, 4). ALM decreased nonlinearly starting in the 4^th^ decade in both genders and in the oldest participants it was reduced by 11% (in women) up to 23% (in men) in comparison to the referent group of 20–39-year-olds. Interestingly ALMI also decreased with increasing age, but only in men. In women, it remained almost steady across all age groups. Similar findings were reported in the CSS [17]. These results are of clinical relevance, as they suggest that interventions aimed at preventing or delaying sarcopenia should take gender-specific differences in muscle mass decline into account. However, we found a disproportion in the number of subjects with low muscle mass when it was assessed by ALM and ALMI. The prevalence of low muscle mass, defined by a *T*-score of 2 ≥ SDs below the young reference group, ranged from 0.13% to 0.89% in women and from 0.3% to 3.05% in men, while a moderate muscle mass deficit (*T*-score between –1 and –2 SDs) ranged from 7.79% to 15.20% in women and from 11.28% to 31.71% in men using ALM and ALMI, respectively. These findings reinforce the importance of using multiple measures of muscle mass and strength to assess sarcopenia risk more comprehensively, as reliance on a single parameter may not provide a full picture of the patient’s muscle health. According to the current definition of sarcopenia, these subjects are classified as having a pre-sarcopenic state [1]. Overall, the frequency of subjects with muscle mass below the young adult reference mean was higher by ALM, particularly in men (Table 5). CSS [17] also reported a higher prevalence of low muscle mass using ALM compared to ALMI (9% vs. 4% in men and 2.5% vs. 1.8% in women with muscle mass less than –2 SDs). Altogether, these findings may suggest that ALM is a more potent discriminator than ALMI in identifying subjects with muscle mass deficits, at least in men. On the other hand, the frequency of low muscle mass in the ABC Study [29] was almost identical in both genders using these two parameters. Further studies are needed to establish the sensitivity of both outcome variables in the assessment of skeletal muscles by DXA in different populations. Using CoPs recommended by EWGSOP2, we found reduced muscle mass in 25.6% (ALM) and 20.3% (ALMI) of women and 8.54% (ALM) and 11.59% of men. In contrast to our cohort, in the EWGSPOP2’s European population, both muscle strength and the level of physical performance were not evaluated as inclusion criteria. Our results seem to confirm the need to develop CoPs for muscle mass in specific, narrow populations, especially since muscle mass CoPs have a greater impact on sarcopenia prevalence than those for grip strength and gait speed [36].

Some earlier studies developed country-specific standards for muscle mass using TLM, or FFM. In DXA, FFM differs essentially from TLM because it is defined as the sum of all non-lipid components of the body (including non-fat elements of the adipose tissue). Although muscles are the main component of FFM, its use as a surrogate measure of muscle mass has been questioned, especially in the elderly and in obese or weight-reduced obese individuals, because in these individuals the contribution of connective tissue to TLM significantly increases [32, 37, 38]. On the other hand, others suggest that both FFM and FFM index (FFMI; FFM/Height^2^) could be an accurate proxy for muscle mass in screening for sarcopenia across a wide range of ages and BMIs [39–41]. It has also been suggested that the FFMI might be a stronger determinant of physical performance than the ALMI [42]. In the present study, TLM and TLMI reached their peak values in 40–49-year-old men and in 60–69-year-old women. After achieving these time points, they decreased or remained steady in older age groups. The mean, sex-specific values of TLMI in our study across investigated age groups were generally comparable with TLMIs in CSS [15] but lower than those found in healthy Italian volunteers [43] and in NHANES reference standards [24]. Different results were obtained from the healthy Caucasian adults living in south-central Italy. Both TLM and TLMI were relatively stable across all age groups in both genders. TLMI in this study was slightly but significantly increased in 45–54-year-old males, while other sex-specific changes in this index with age were not significant [42]. In the adult Kenyan population aged over 50 years, FFMI decreased starting at the age of 60, both in men and women [40]. In 5635 apparently healthy adults from a mixed, non-randomly selected Caucasian population in Switzerland aged 19-98 years, Schutz et al. [37] observed no significant changes in FFMI performance across all age groups, while fat mass increased with age. However, there were significant differences between these studies in BMI and the amount of fat mass.

Our study has some limitations. Firstly, the population samples analyzed in some age groups were modest, particularly in the 30–39, 40–49, 50–59, and ≥ 80-year groups, with men being underrepresented. It was mainly caused by the fact that we recruited only apparently healthy individuals, and they were aware that they would receive no direct benefits from their participation in the study. The prevalence of comorbidities (cardiovascular diseases, malignancies, chronic arthritis, mental disorders, etc.) increases with age; hence, the availability of healthy subjects of advanced age who choose to participate in clinical research because they want to help others and contribute to advancing science is objectively limited.

Furthermore, the underrepresentation of men in our study reflects a broader trend in musculoskeletal research. It is notable that men, particularly those in older age groups, are less inclined to participate in health-related studies, particularly those focusing on conditions such as sarcopenia and other musculoskeletal disorders. This gender disparity has been well-documented in previous research, and several factors may contribute to this phenomenon, including lower health-seeking behaviors and cultural norms that discourage men from engaging in preventive health measures [44–46]. Moreover, according to the latest official estimates, life expectancy at birth in 2021 in Poland was 71.8 years for men and 79.7 years for women, which might explain the lower representation of men participating in this study, particularly in the older age groups. Secondly, in our cohort, we did not evaluate smoking and alcohol use. Recent reports strongly suggest that higher levels of alcohol consumption [47] and cigarette smoking [48] could have detrimental effects on muscle mass, especially in middle- and older-aged men and women.

In future research, it would be beneficial to include detailed assessments of these lifestyle factors. Incorporating validated questionnaires such as the Alcohol Use Disorders Identification Test (AUDIT) and the Fagerström Test for Nicotine Dependence could provide a more comprehensive understanding of how smoking and alcohol consumption interact with muscle mass indices and influence the development of sarcopenia. By integrating these factors, future studies can offer a more nuanced analysis of the multifactorial nature of muscle decline in aging populations.

In conclusion, while our study provides valuable insights into muscle mass indices in a healthy Polish population, future research should focus on addressing these limitations by increasing sample sizes, particularly among older men, and considering the impact of lifestyle factors such as smoking and alcohol consumption on muscle health.

## CONCLUSIONS

We have developed the reference values for ALM and TLM using data from the Hologic Horizon DXA system in a large cohort of healthy Polish men and women aged 20–89 years. This is the first study in which a healthy population was defined using additional criteria, including normal muscle strength and normal physical performance. Therefore, the proposed reference standards for lean mass measures may help improve the validity of muscle wasting assessments. It seems that the CoPs for determining low muscle mass without considering the specificity of the population, could lead to may lead to over- or under-diagnosis of sarcopenia, and due to this it seems important to create lean mass indices reference values specific to narrow populations.
